# Retraction of Adult neural stem cells expressing IL-10 confer potent immunomodulation and remyelination in experimental autoimmune encephalitis

**DOI:** 10.1172/JCI198608

**Published:** 2025-09-16

**Authors:** Jingxian Yang, Zhilong Jiang, Denise C. Fitzgerald, Cungen Ma, Shuo Yu, Hongmei Li, Zhao Zhao, Yonghai Li, Bogoljub Ciric, Mark Curtis, Abdolmohamad Rostami, Guang-Xian Zhang

Original citation: *J Clin Invest*. 2009;119(12):3678–3691. https://doi.org/10.1172/JCI37914

Citation for this retraction: *J Clin Invest*. 2025;135(18):e198608. https://doi.org/10.1172/JCI198608

Errors made using image editing software were identified in Figure 6B and Supplemental Figure 4B. The Editors are retracting the article due to misrepresentation of data.

Guang-Xian Zhang dissents from the Journal’s decision to retract. The remaining authors abstained from commenting or could not be reached.

## Version 1 09/16/2025

Electronic publication

